# Supplementary material for: The interactome of intact mitochondria by cross-linking mass spectrometry provides evidence for coexisting respiratory supercomplexes
Source: Mol Cell Proteomics. 2017 Dec 8;17(2):216–32. doi: 10.1074/mcp.RA117.000470 (PMC5795388; doi:10.1074/mcp.RA117.000470)
Supplement: Supplemental Data [file supp_RA117.000470_133922_0_supp_23978_fzffwf.zip › spectra_annotation/mito_DR_spectra_annotation/79-1-15-1-10-1.pdf]

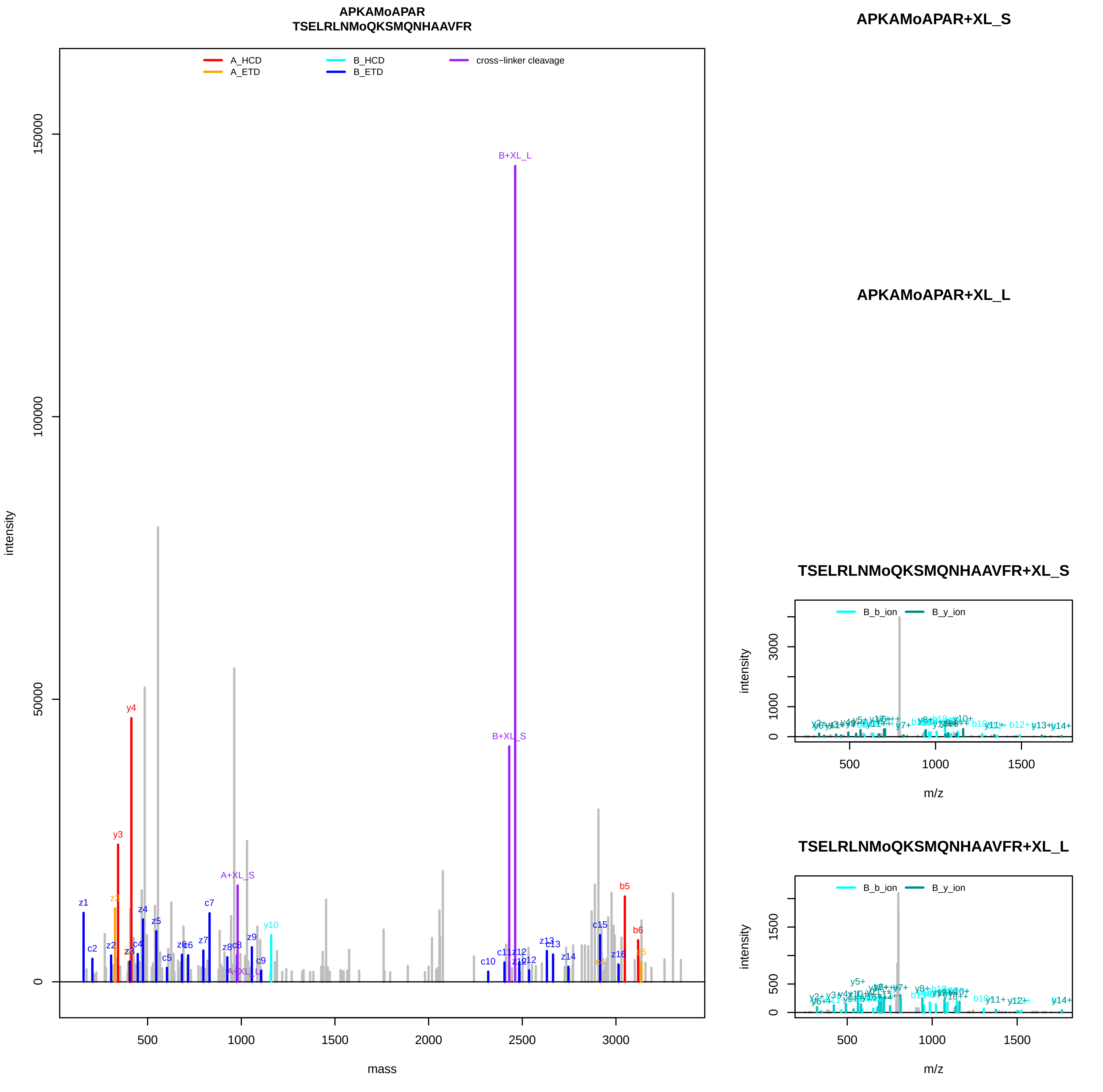

APKAMoAPAR
TSELRLNMoQKSMQNHAAVFR
A_HCD
A_ETD
B_HCD
B_ETD
cross−linker cleavage
intensity
mass
0
50000
100000
150000
500
1000
1500
2000
2500
3000
B+XL_L
B+XL_S
A+XL_S
A+XL_L
y4
y3
b5
b6
y10
z1
c2
z2
z3
c4
z4
z5
c5
c6
z7
c7
z8
c8
z9
c9
c10
z13
c13
z14
c15
z16
APKAMoAPAR+XL_S
APKAMoAPAR+XL_L
TSELRLNMoQKSMQNHAAVFR+XL_S
TSELRLNMoQKSMQNHAAVFR+XL_L
B_b_ion
B_y_ion
intensity
m/z
0
1000
2000
3000
500
1500
